# A review of antibiotic prophylaxis for traveler’s diarrhea: past to present

**DOI:** 10.1186/s40794-018-0074-4

**Published:** 2018-11-07

**Authors:** Ajib Diptyanusa, Thundon Ngamprasertchai, Watcharapong Piyaphanee

**Affiliations:** Department of Clinical Tropical Medicine, Faculty of Tropical Medicine, Mahidol University, 420/6 Ratchawithi Road, Ratchathewi, Bangkok, 10400 Thailand

**Keywords:** Antibiotic, Prophylaxis, Prevention, traveler’s diarrhea

## Abstract

As there is rapid increase in international travel to tropical and subtropical countries, there will likely be more people exposed to diarrheal pathogens in these moderate to high risk areas and subsequent increased concern for traveler’s diarrhea. The disease may appear as a mild clinical syndrome, yet a more debilitating presentation can lead to itinerary changes and hospitalization. As bacterial etiologies are the most common causative agents of TD, the use of antibiotic prophylaxis to prevent TD has been reported among travelers for several years. The most common type of antibiotic used for TD has changed over 50 years, depending on many influencing factors. The use of antibiotic prophylaxis for TD prevention in travelers is still controversial, mainly because of difficulties balancing the risks and benefits. Many factors, such as emerging drug resistance, side effects, cost and risk behavior need to be considered. This article aims to review antibiotic prophylaxis from the 1950s to 2000s, to describe the trend and reasons for different antibiotic use in each decade. We conclude that prophylactic antibiotics should be restricted to some high-risk travelers or short-term critical trips.

## Introduction

International travel is rapidly increasing, with 1.2 billion travelers in 2016, and Asian and Pacific regions being particularly popular [1]. Traveler’s diarrhea (TD) is one of the most common travel-related illnesses among short-term travelers to low- and middle-income countries [2, 3]. Incidence of TD ranges from 2 to 57%, for different traveler characteristics and destinations [4–10]. High risk destinations for contracting TD include most countries in Asia, the Middle East, Africa, Mexico, and Central and South America [11, 12]. Most TD usually results in mild symptoms and is self-limiting [13, 14], however clinical symptoms can be severe and cause several issues, including disruption to travel, or long-term effects and hospitalization [15, 16].

### Etiology of traveler’s diarrhea

The causative pathogens of TD vary in each region, but bacteria are the most common, followed by viruses and protozoa. In Latin America and the Caribbean, the most common pathogens causing TD are enterotoxigenic *Escherichia coli* (ETEC) and enteroaggregative *E. coli* (EAEC) [17, 18]. In addition, ETEC has been documented as the most common pathogen in travelers returning from African countries [19]. As well as ETEC, *Campylobacter, Giardia* and *Shigella* have frequently been reported to cause TD in travelers in the Indian subcontinent [20, 21]. Interestingly, *Campylobacter* is the most common pathogen isolated from travelers returning from Southeast Asia [17, 22]. Increasing reports of norovirus in travelers returning from multiple regions of the world are of concern, as it is an important cause of TD [23].

Travelers to remote areas far from medical facilities are often advised to take antibiotics when symptoms of TD develop [24]. In contrast, some high-risk groups, such as immunocompromised travelers, might prefer to take antibiotics prophylactically to prevent TD. There have been many reports that bacterial etiology is the most common cause of TD, therefore antibiotic use might be the most effective method of prevention [17–22]. It has been reported previously that approximately 15% of travelers take antibiotics to prevent TD [25]. However, the use of antibiotic prophylaxis for TD prevention in travelers is still controversial, mainly because of the challenge of managing risks and benefits [26].

### Antibiotic prophylaxis

Traditionally, standard pre-travel consultations include advice to “boil it, cook it, peel it, or forget it” to prevent TD [11]; nevertheless, studies have reported that even travelers who follow these rules may develop TD [27, 28]. Therefore, the use of antibiotic prophylaxis for travelers could be considered, to decrease the pathogen burden and prevent long-term morbidity [29, 30]. The prophylactic antibiotic of choice has been changing over the last few decades, as resistance patterns developed [31]. According to previously published randomized controlled trials (RCTs), antibiotic efficacy varies from 28% up to 72% [32–36], for different types of antibiotics and traveler’s destinations.

## Method

We searched the PubMed database for publications on the protective efficacy of antibiotics as chemoprophylaxis for TD. Search terms included *antibiotics*, *travel, diarrhea, neomycin, furazolidone, doxycycline, trimethoprim/sulfamethoxazole, erythromycin, mecillinam, bicozamycin, ciprofloxacin, norfloxacin, azithromycin* and *rifaximin*. All search articles yielded 616 in numbers. Inclusion criteria were papers written in English and related to the use of antibiotics as prophylaxis for TD. Overall, 27 studies were included in this review, to illustrate the trend of antibiotic use in the prevention of TD over the past 50 years (as shown in Table 1).

**Table 1:** Selected publications showing prophylactic antibiotic use and its protection rate against TD in chronological order from oldest to newest

Publication year	Drug name	Participants studied	Protection rate (%)	Reference
1960s				
1962	Neomycin	305	32	[39]
1962	Phthalylsulfathiazole	336	50	[39]
1970s				
1978	Doxycycline	39	86	[43]
1979	Doxycycline	50	83	[44]
1980s				
1981	Doxycycline	46	27	[45]
1981	Erythromycin	48	71	[54]
1982	TMP/SMX	147	71	[49]
1983	TMP/SMX	87	95	[50]
1983	Mecillinam	74	75	[56]
1983	Doxycycline	145	81	[48]
1984	Doxycycline	63	68	[46]
1984	Doxycycline	44	59	[47]
1985	Mecillinam	32	66	[57]
1985	Bicozamycin	30	100	[59]
1986	Norfloxacin	115	88	[63]
1987	Norfloxacin	115	70	[64]
1989	Ciprofloxacin	59	94	[61]
1990s				
1990	Norfloxacin	222	93	[65]
1994	Ciprofloxacin	21	100	[62]
1994	Ciprofloxacin	99	84	[51]
1994	TMP/SMX	87	51	[51]
1996	Azithromycin	231	81	[72]
2000s				
2005	Rifaximin	210	72	[33]
2010	Rifaximin	95	67	[34]
2010	Rifaximin	201	58	[35]
2011	Rifaximin	98	28	[32]
2013	Rifaximin	239	48	[36]

## Results

### Enterovioform, neomycin, phthalylsulfathiazole and furazolidone

The first attempt to use antibiotics to prevent TD was in the late 1950s, using enterovioform and neomycin [37]. Since the use of enterovioform—an iodochlorhydroxyquin—was associated with myelo-optic neuropathy, the drug was withdrawn from the market [38]. In the early 1960s, a double-blind study was conducted in American college students in Mexico City, to compare a placebo with low doses of neomycin sulfate and a sulfonamide called phthalylsulfathiazole [39]. Low protective efficacy was observed in neomycin sulfate, yet phthalylsulfathiazole halved the incidence of TD. There were no adverse drug reactions reported during the 14-day study. Another trial using neomycin-trisulfamide, Streptotriad (a combination of streptomycin and triple sulphonamides), or a placebo was conducted in British Airways personnel and their families, traveling to different countries worldwide, for up to 3 weeks [40]. Both drugs resulted in a low protection rate against TD. Comparable low protective efficacy was reported in the use of a nitrofuran derivative furazolidone for TD prophylaxis in 223 Royal Air Force pilots [41].

### Doxycycline

During the late 1970s, doxycycline was used initially. The role of doxycycline for the prevention of TD was supported by research that showed the drug was highly effective against ETEC as major cause of TD, had long-acting properties and minimal adverse events [42]. Daily administration of 100 mg of doxycycline for 3 weeks was up to 86% effective for TD prevention among American Peace Corps Volunteers, deployed to multiple regions of the world [43, 44]. Unfortunately, lower protective efficacy was observed over the proceeding 5 years, because of high proportions of tetracycline-resistant ETEC [45–47]. Hence, a trial using a higher dose of doxycycline (200 mg per day) was conducted. The results showed a significant reduction in the incidence of TD, but 12% of cases reported gastric upset in the doxycycline group [48]. Today, because of the high risk of antimicrobial resistance and potential side effects from using doxycycline, no guidelines recommended its use.

### Trimethoprim/sulfamethoxazole

During the late 1970s to early 1980s, some studies showed that the use of trimethoprim/sulfamethoxazole (TMP/SMX) prophylaxis resulted in a significantly higher reduction of TD incidence in college students traveling to Guadalajara, Mexico when compared with a placebo and TMP alone [49, 50]. Administration of 160 mg TMP and 800 mg SMX twice daily for 3 weeks yielded a protective efficacy of 71% [49]. Another study showed that the same daily dosage of TMP/SMX for 14 days resulted in 95% protection against TD [50]. Emerging drug resistance was a concern, as a higher resistance pattern for *E. coli* had been observed since the early 1980s [50–52]. Dermatologic adverse reactions, especially skin rashes, were also reported.

### Erythromycin, mecillinam and bicozamycin

**Fig. 1:**
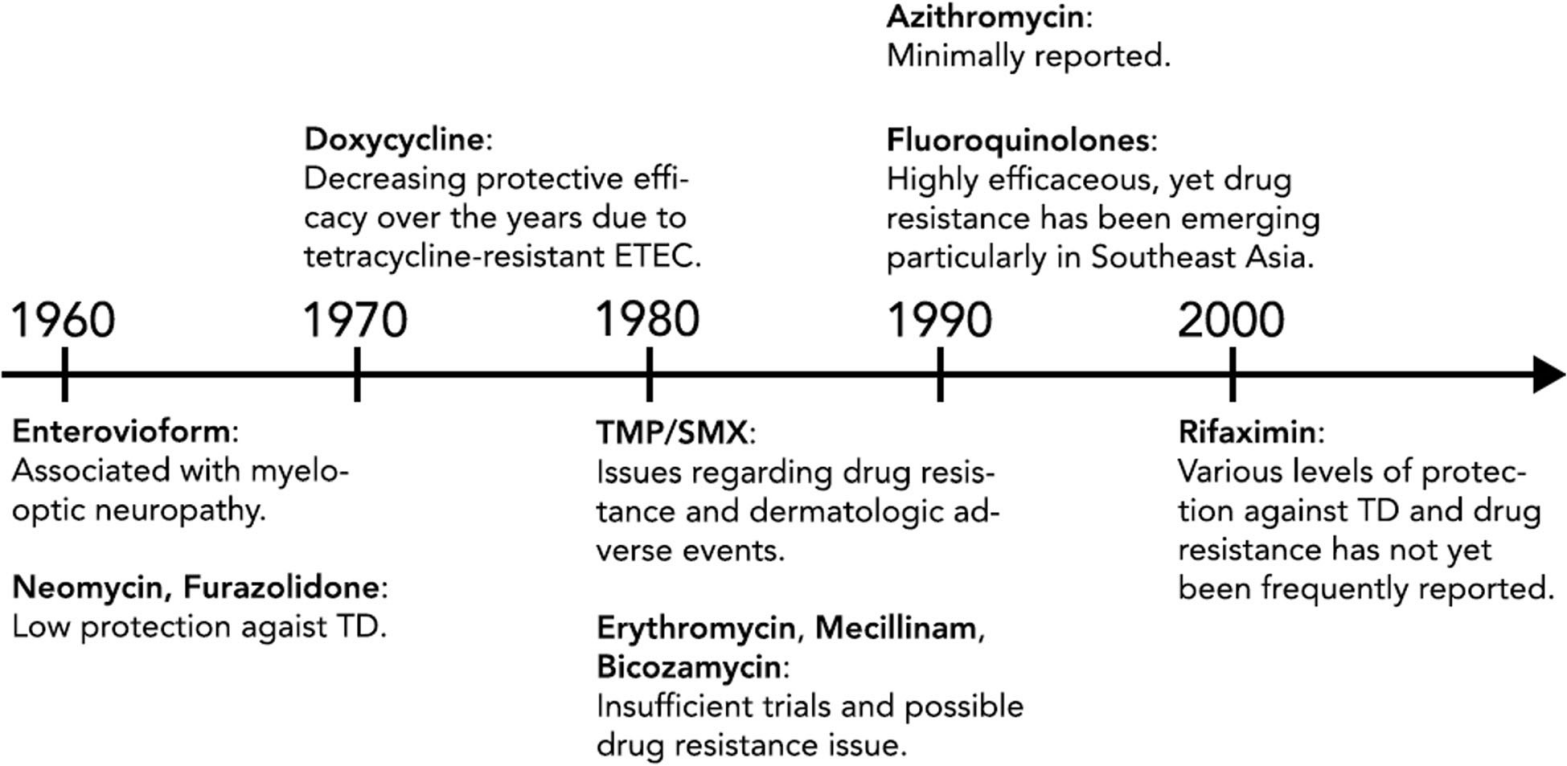
Timeline of prophylactic antibiotic use against TD in chronological order

Emerging drug resistance resulted in the need to find new regimens for the chemoprophylaxis of TD (Fig. 1). In early 1980s, trials in TD prevention using erythromycin, mecillinam and bicozamycin were conducted. This is because erythromycin had been reported to eliminate Enterobacteriaceae from human fecal flora without evidence of recolonization [53]. A placebo-controlled RCT on American travelers to Mexico, using 1 g erythromycin during travel showed a protective efficacy of 71% against TD [54]. Furthermore, a non-absorbable antibiotic, mecillinam, was also identified as having selective bactericidal activity against Enterobacteriaceae [55]. A trial using 200 mg of mecillinam for 25 days as prophylaxis for TD was conducted in adult travelers visiting Asia, Africa or Latin America, resulting in 75% protection against TD [56]. A similar protection rate was reported among Danish travelers to Mexico [57]. However, it should be noted that etiology of most TD cases remained unknown. Bicozamycin, or bicyclomycin, has been found to have similar activity against Enterobacteriaceae [58]. Administration of 500 mg of bicozamycin four times daily for 21 days, to adult US citizens traveling to Guadalajara, Mexico, showed high protection against TD [59]. As well as the inconvenience of frequent pill taking for travelers, resistance of bicozamycin had also been detected [59]. The drug was not further developed for human use.

### Fluoroquinolones: ciprofloxacin and norfloxacin

Discovery of quinolone antibiotics gave researchers hope to prevent traveler’s diarrhea, as this group of antibiotics is highly effective against gram-negative pathogens including Enterobacteriaceae [60]. The early clinical trials using ciprofloxacin and norfloxacin were conducted during the late 1980s and early 1990s [51, 61–65]. These two drugs have been shown to be highly effective in the prevention of TD, with less frequent adverse events. Administration of 500 mg of ciprofloxacin daily in two studies demonstrated protective efficacies of 84% and 94% [51, 61]. To compare fluoroquinolones with previous antibiotics, ciprofloxacin provided a higher protection rate, compared with TMP/SMX (84% versus 51%, respectively); however, the Asian region was not included in this study. A double-blind randomized trial using 250 mg of ciprofloxacin was conducted in a Himalayan expedition team in 1994, yet the protection rate for TD was not clearly reported [62]. Further studies using norfloxacin with a dose of either 400 mg daily or 200 mg twice daily showed 70–93% protection against TD [63–65].

Antibiotic resistance to fluoroquinolones, especially in *Campylobacter* species has been reported, particularly in Southeast Asia [66–68]. Broad use of fluoroquinolones in both humans and in animals along with chromosomal mutations in drug’s target enzymes and efflux systems, were sufficient to cause important levels of clinical resistance [69, 70]. In addition to its ease in drug resistance development, the risk of tendinitis/tendinopathy, QT prolongation, and delirium could limit the use of fluoroquinolones for TD prevention [2, 71].

### Azithromycin

The protective effect of azithromycin against dysenteric diarrhea has been accidentally discovered during malaria chemoprophylaxis efficacy trial in Kenya in 1995. A total of 231 volunteers were divided into 4 arms: azithromycin 250 mg daily, azithromycin 1000 mg weekly, doxycycline 100 mg daily, or placebo. Result showed that only 2.6% of volunteers in the daily or weekly azithromycin groups developed dysentery, while 13.9% of volunteer in doxycycline or placebo group developed dysentery in the same period, so the estimated protective efficacy of azithromycin was 81% [72]. Unfortunately, although azithromycin is very well known for its efficacy in treatment of TD; the study of its use as chemoprophylaxis against TD is rarely reported.

### Rifaximin

Side effects and development of resistance among extraintestinal bacteria means the use of absorbable (systemic) antibiotics to prevent TD has been discouraged [73]. In the 1990s, an antibiotic called rifaximin was discovered and shown to be effective for the prevention of TD without causing significant adverse effects. Rifaximin is a rifamycin derivative, which is poorly absorbed and able to reach high concentrations in the intestinal lumen [74]. Rifaximin has also been shown to effectively prevent shigellosis [75].

Studies in short-term travelers to Mexico, using various regimens of rifaximin for 14 days, demonstrated protective efficacies ranging from 28 to 72% [32, 33, 35]. Another study showed that the use of a higher dose of 1100 mg rifaximin for 14 days resulted in 67% protection against TD in military personnel deployed to Turkey [34]. The latest trial using 200 mg of rifaximin, twice daily from the day of departure to 7 days post-return in adult travelers visiting South Asia and Southeast Asia, showed a protective efficacy of 48% [36].

Low to moderate efficacy of rifaximin has been observed in several studies, however the majority of the studies [33–36] did not report the resistance pattern of the causative pathogen. Therefore, it remains unclear whether the low protection level was because of rifaximin resistance. One study reported unusually high rifaximin resistant *E. coli* in several countries [76].

## Factors to consider and recommendations

Several factors need consideration when prescribing prophylactic antibiotics for the prevention of TD. Individual risk assessment, including type of population (Table 2), travel purpose and itinerary will be helpful for making prescribing decisions [31]. All the RCTs reported here were conducted in travelers from high-income countries. Applying similar regimens to travelers from low- or middle-income countries may not yield similar efficacy. Furthermore, the incidence of TD in Asian travelers only ranges from 1.6 to 8%, therefore in general travelers, reducing the incidence of TD with antibiotic prophylaxis may not be beneficial [7–9]. Immunocompromised travelers, those taking important trips, or visiting remote locations with a lack of medical provision, may be targeted for antibiotic prophylaxis [26, 31]. As up to 50% of immunocompromised travelers will experience a gastrointestinal illness including diarrhea [77], it is advisable for this group of travelers to take prophylactic antibiotics; otherwise they will be at risk of more severe illness and hospitalization during travel [78]. In addition, drug side effects should also be considered when making prescribing decisions [79].

**Table 2:** Types of population that might benefit for TD chemoprophylaxis

High-risk travelers	
- Elderly	
- Immunocompromised travelers	
- Travelers with low gastric acidity	
- Travelers with chronic gastrointestinal diseases	
Travelers who take very important/critical trips	
- Very important governmental officials	
- Diplomats	
- Athletes	
- Professional musicians	

Host microbiome may be an important factor involved in protecting travelers from TD. Alterations in native gut microbiome, known as dysbiosis, are known to be associated with travel [80]. Ingestion of antibiotics could affect gut microbiome by several mechanisms such as decrease the diversity, expansion of the resistant strain, loss of the keystone species that support normal ecology [81]. All of these factors can increase the susceptibility of TD. Unfortunately, even short term antibiotics exposure could disrupt the gut microbiome for a year or more and repeated exposure could delay its recovery [81]. Apart from the use of antibiotic, several factors including change of sleeping pattern, exposure to local diet and water or ingestion of antibiotics during the trip can disrupt the gut microbiome [80, 82].

Other factors may affect the efficacy of prophylactic antibiotics, such as the predominant pathogens present in each geographical region and their pattern of antibiotic resistance [83]. Drug resistance has emerged over several years, from ETEC resistance to tetracyclines and TMP/SMX, followed by fluoroquinolone-resistant *Campylobacter* strains from Southeast Asia. Hence, fluoroquinolones should not be prescribed as prophylactic antibiotic for TD in travelers traveling to Southeast Asia. Unfortunately, data for antibiotic resistance are unavailable in some regions of the world, especially in low- and middle-income countries [84]. The current data for rifaximin suggest it could be a suitable prophylactic treatment for TD, as there is no evidence of resistance developing.

Although TD is perceived as a self-limiting disease, it represents a substantial socioeconomic burden from traveler’s perspective [85]. This illness may affect not only a change in lifestyle induced by the illness itself, but also expenses for medication and medical services. Cost-benefit analysis, by comparing the cost of antibiotic prophylaxis and treatment involving loss of productivity, may also be a useful approach [79, 86].

Current recommendations suggest that antibiotic prophylaxis for TD may be prescribed selectively in some travelers, especially in high-risk short-term travelers [26]. Rifaximin is preferred to other antibiotics because of its poor absorption, reducing the risk of development of resistance in extraintestinal bacteria [26]. However, it is important to note that not all TD is caused by bacteria, so even if we had ideal antibiotics, these would not prevent TD caused by viruses or protozoa.

## Conclusions

Trends in antibiotic use for TD prevention have been changing over several decades. Prophylactic antibiotic prescribing for TD should always include an individual risk assessment, including type of traveler, their destination, travel purpose, itineraries, drug side effects, and cost-benefit analysis. The global increase in antibiotic resistance limits the choice of antibiotics. In the past, doxycycline, TMP/SMX or fluoroquinolones may have been effective for TD prevention; however currently, minimally-absorbed rifaximin is recommended for travelers.

## Abbreviations

EAEC: Enteroaggregative *Escherichia coli*
ETEC: Enterotoxigenic *Escherichia coli*
RCT: Randomized controlled trial
TD: Traveler’s diarrhea
TMP/SMX: Trimethoprim/sulfamethoxazole
